# Type 2 Diabetes and the Aging Pancreatic Beta Cell

**DOI:** 10.18632/aging.100350

**Published:** 2011-06-28

**Authors:** Uma Gunasekaran, Maureen Gannon

**Affiliations:** ^1^ Departments of Medicine; ^2^ Molecular Physiology and Biophysics; ^3^ Cell and Developmental Biology, Vanderbilt University, Nashville, TN; ^4^ VA Medical Center, Nashville, TN; ^5^ Division of Diabetes, Endocrinology and Metabolism, 2213 Garland Avenue MRBIV 7465, Nashville, TN 37232-0475

## Abstract

The incidence of and susceptibility to Type 2 diabetes increases with age, but the underlying mechanism(s) within beta cells that contribute to this increased susceptibility have not been fully elucidated. Here we review how aging affects the proliferative and regenerative capacity of beta cells and how this impacts beta cell mass. In addition we review changes that occur in beta cell function with age. Although we focus on the different rodent models that have provided insight into the characteristics of the aging beta cell, the limited knowledge from non-rodent models is also reviewed. Further studies are needed in order to identify potential beta cell targets for preventing or slowing the progression of diabetes that occurs with age.

## INTRODUCTION

The incidence and prevalence of Type 2 diabetes increases with age [1, 2]. It now affects approximately 18-30% of the elderly population in the United States [3, 4]. The underlying mechanism(s) behind why diabetes is increasing in the elderly is still not clearly understood. It has been hypothesized that insulin resistance increases with age due to increased adiposity, decreased lean muscle mass, changes in dietary habits, and reduced physical activity [5]. However, it has been shown that these factors alone do not account for age-related glucose intolerance [1]. Many human studies, some of which are summarized below, have tried to clarify the mechanism by which age-related glucose tolerance develops but have had contradictory results.

Early studies on the increase in peripheral insulin resistance due to age alone yielded inconclusive results [6]. For example, in one study comparing young (ages 18-36) and older (ages 57-82) men, subjects underwent frequent measurement of plasma glucose and insulin levels during an intravenous glucose tolerance test with arginine potentiation [7]. This study found that there was a significant decrease in the glucose clearance rate in the older subjects despite elevated plasma insulin levels in this population compared to the younger men. These data imply that insulin sensitivity decreases with age. In contrast, a different study of young (ages 19-36) and older (ages 47-73) men used a hyperinsulinemic clamp model to measure plasma glucose clearance. In this study, plasma glucose clearance was found to be dependent on the waist-to-hip ratio and not age: higher waist-to-hip ratios were associated with impaired plasma glucose clearance, implying that it is fat distribution that portends insulin resistance rather than age alone [8].

Studies on the age-related effects on the beta cell have also yielded inconsistent results. A retrospective analysis of the European Group for the Study of Insulin Resistance database revealed a 25% decline in the insulin delivery rate (calculated as the sum of the clamp-derived posthepatic insulin clearance rate and fasting plasma insulin concentration) from age 18 to 85 [1]. This study controlled for body mass index (BMI), fasting plasma glucose, and insulin sensitivity in both men and women. These results suggested that beta cell function declines with age. In contrast, in a study of young (ages 23-25) and older (ages 64-66) adults using a hyperglycemic clamp technique, defects in beta cell function in older individuals were only observed with pre-existing impaired glucose tolerance (IGT) or Type 2 diabetes; normoglycemic older individuals had a similar insulin response to hyperglycemia as their younger counterparts [9]. The results of this study suggest that there is not necessarily an overall age-related decrease in beta cell function but may be observed only in the setting of IGT or frank diabetes.

**Figure 1:**
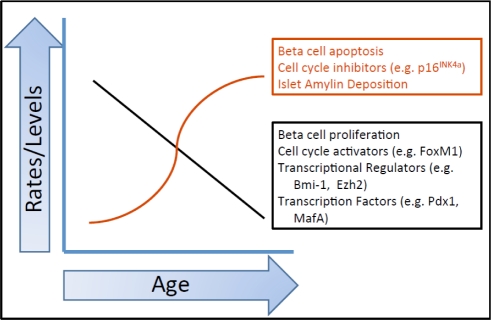
Summary of the effects of age on various beta cell parameters. Multiple factors influence the beta cell as it ages. Each of the factors listed here is discussed in more detail in the text. This graph is a representation of how these parameters change with age. The actual kinetics of each of these changes has not been fully elucidated.

These macroscopic studies are difficult to interpret because glucose intolerance can develop from a combination of many factors and controlling for every possible influence is impossible. Therefore, studies have now focused on the effect of aging on the beta cell, specifically on insulin secretion, beta cell mass, and the proliferative and regenerative capacity of the beta cell. This review will examine early theories on how beta cell function decline with age as well as explore what is known about beta cell proliferation, apoptosis, and the role of regeneration and neogenesis in the aging beta cell. Finally, the factors that maintain beta cell function will be reviewed in relation to age. It is important to note that there is currently limited information in the field on aging in the non-diabetic human and rodent models, but what is known will be reviewed here (Figure 1). Changes in body mass and insulin resistance and the effect that these have on peripheral tissues, such as muscle and adipocytes is another topic in itself and will not be a subject of this review.

### Early theories on why beta cell function declines with age

Early hypotheses on possible causes for the decline in beta cell function with age considered theories surrounding alterations in glucose oxidation as well as alterations in potassium efflux and levels of calcium ions. In the beta cell, glucose oxidation results in the increased ATP production required for insulin secretion. Therefore, a reduction in glucose oxidation rates with aging would result in reduced insulin secretion. Indeed, some studies have shown that older animals have reduced rates of glucose oxidation [10]. However, subsequent studies revealed that older rats actually show an increased rate of both glucose oxidation and insulin secretion, suggesting that this is a potential mechanism by which beta cells attempt to overcome age-associated peripheral insulin resistance [10].

There has been some discussion that age affects the potassium and calcium channels involved in insulin secretion. Normally, elevated glucose concentrations induce beta cells to inhibit potassium efflux, resulting in increased calcium influx through voltage-dependent calcium channels. The increase in intracellular calcium induces insulin exocytosis. In a study of potassium channels comparing 3 month to 24 month old rats, a significant potassium efflux even after high glucose stimulation was observed in older rats, indicating that the normal inhibition of the K_ATP_-channel is lost with age [11]. These data indicate that age-related alterations in beta cell function could be due to changes in glucose oxidation and ion channel function.

### Beta cell proliferation declines with age

The regenerative capacity of most organs decreases with age. Older rats exhibit reduced proliferative capacity in the form of reduced muscle and bone mass, defective tissue repair, and thinning of skin [12]. Specifically, this may be due to an age-related reduction in expression of the Forkhead Box M1 (FoxM1) transcription factor [12]. FoxM1 regulates genes involved in cell cycle regulation and cell division. It is highly expressed in proliferating cells, and expression declines in most cell types with age, including pancreatic islets [13]. Genetic inactivation of the *Foxm1* gene throughout the pancreatic epithelium results in reduced postnatal beta cell proliferation [13-15].

Beta cell proliferation is also reduced in humans with age [16, 17]. A small study of pancreata from twenty non-diabetic organ donors aged 7 to 66 showed a decline in beta cell replication with age [16]. The decline in beta cell replication was directly associated with a decrease in expression of the pancreatic and duodenal homeobox 1 (pdx1) [16], a transcription factor, known to be important for beta cell replication [18] and will be discussed further later in this paper. In another study, a review of 124 pancreata from obese, diabetic, and lean individuals aged 61-83 at autopsy, showed that in this population, there was also a low frequency of beta cell replication [17].

### Cell cycle activators and inhibitors are targets for promoting beta cell replication.

Investigating the expression and function of different cell cycle activators and inhibitors in the beta cell could elucidate mechanisms by which beta cell replication can be promoted to enhance beta cell mass (and subsequently possibly function), especially in the setting of diabetes. Beta cells express most of the known cell cycle inhibitors, including p16^INK4a^, p18^INK4c^, p21^CIP1^, p27^Kip1^, p53, and Rb [19]. In contrast, there is much less redundancy of cell cycle activators in the beta call. For example, rodent beta cells express only Cdk4 (cyclin-dependent kinase 4) and not Cdk6, whereas most other cell types express both of these closely related proteins [20].

Mouse beta cells express all three D cyclins, D1, D2, D3, but the mRNA expression of D2 is significantly higher than both D1 and D3 with only D2 detectable by immunohistochemistry [21]. The D cyclins promote the passage of cells past the G phase checkpoint and in conjunction with the cyclin-dependent kinases [22]. Loss of a single cell cycle inhibitor does not accelerate beta cell cell cycle progression, whereas loss of multiple inhibitors enhances beta cell proliferation [23] [24] [25]. For example, beta cell-specific inactivation of the *Rb* gene has minimal effect on beta cell replication rates, pancreatic insulin content, and beta cell mass [26]. Conversely, loss of a single cell cycle activator leads to profound defects in beta cell replication and beta cell mass. Specifically, loss of Cdk4 leads to a reduction in mRNA levels of *insulin I, insulin II, islet amyloid polypeptide*, and *Glut2* as well as decreased beta cell area (from deformed and smaller-size islets) [27]. Additionally, loss of the transcription factor FoxM1 in the pancreatic epithelium results in decreased postnatal beta cell mass [13], at least in part due to increased nuclear p21 levels and decreased Cdk2 activation via reduced Cdc25A phosphatase expression [28]. This phenomenon points to the potentially greater importance of regulating cell cycle “brakes” or inhibitors rather than solely trying to increase expression of a particular “accelerator” or activator.

#### Cell cycle activators

As mentioned above, D cyclins and cyclin-dependent kinases promote progression from G phase to S phase. Loss of cyclin D1 and D2 in beta cells has no effect on islet size or number, indicating that these cell cycle activators are not important to embryonic beta cell development. However, cyclin D2 null mutant mice develop diabetes by 9-12 months, which suggests that it is critical to adult beta cell expansion [21]. Studies by He et al. indicate that phosphorylation of threonine 280 (T280) within cyclin D2 limit its stability. Transgenic mice expressing a mutant form of cyclin D2 under the insulin promoter, where T280 was mutated to alanine allowing for increased levels of D2 expression, showed increased beta cell mass at 18-21 months [30]. Transgenic mice over-expressing cyclin D1 displayed increased beta cell proliferation, interestingly without inducing tumor formation [29]. Cyclin D3 levels are nearly undetectable in mouse islets and no studies have been performed to examine the impact of an absence of cyclin D3 in the beta cell. In contrast, cyclin D3 is highly expressed in the human beta cell, with only minimal cyclin D1 and D2 levels noted. Interestingly, cyclin D3 over-expression (in isolated human islets), especially in combination with cdk6, induced the greatest increase in beta cell proliferation when compared with over-expression of other cyclins (cyclins D1,2,3 and cdk 4 and 6) [31].

Through their interaction with the cyclins, Cdk's inactivate cell cycle inhibitors allowing for cell cycle progression [32]. Cdk4 null mutant mice show no impairment in embryonic beta cell development, but postnatally they have a significant reduction in beta cell mass compared to controls. In fact, by age 17 weeks, beta cell mass of Cdk4 null mutant mice is only 10% of that of age-matched controls. Null mutants have a 3.5-fold reduction in BrdU incorporation as well, but, interestingly, only beta cells, and not other pancreatic endocrine cell types, are affected [20]. Additionally, mice were generated with a mutation in the Cdk4 protein such that it could no longer be down-regulated by p16^INK4a^. In this model, there was a 7-10 fold increase in islet cell area with no significant change in glucose tolerance when compared to wild-type littermate controls aged ≥ 3 months [27]. Cdk2 is also present in the mouse islet [33], but its specific role in beta cells has not yet been elucidated. Cdk6, though important to the cell cycle in other cell types, is not expressed in mouse islets [33] but is very effective in driving beta cell replication in human islets [31]. The effect of age on cyclin D and Cdk levels has not yet been examined.

#### Cell cycle inhibitors

**Figure 2:**
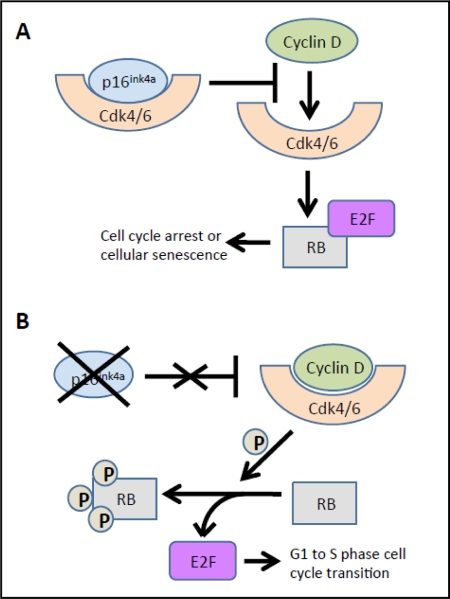
Effects of p16^Ink4a^ on Cdk4/6. **(A)** The p16^Ink4a^ cell cycle inhibitor sequesters Cdk4 or Cdk6, preventing interactions with cyclin D proteins, and thus phosphorylation of pRB. Hypophosphorylated pRB sequesters the E2F transcription factor, thus thus inihibiting cell cycle progression. **(B)** In the absence of p16^Ink4a^, cyclin D forms a productive complex with either Cdk4 or Cdk6 and phosphorylates RB. This phosphorylation releases the E2F transcription factor, facilitating the G1 to S phase cell cycle transition. Thus, in the presence of elevated *p16^Ink4a^*, such as with aging, there is cell cycle arrest and cellular senescence. Adapted from [32]

**Figure 3:**
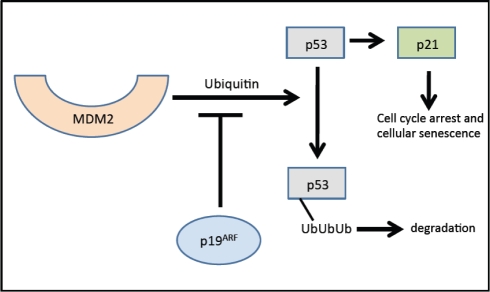
Effects of p19^Arf^ on the cell cycle. In the absence of p19^Arf^, p53 is ubiquitinized and subsequently degraded by the proteosome. p19^Arf^ inhibits the ubiquitin-modulated effect of p53 by MDM2. p19^Arf^ inhibits ubiquitin-mediated p53 degradation resulting in induction of p21 and subsequently cell cycle arrest. Adapted from [75] and [76]

Evidence from multiple labs has demonstrated that with age, beta cells show decreased expression of cell cycle activators (e.g., FoxM1 mentioned above) with simultaneous increases in expression of cell cycle inhibitors. For example, the p16^Ink4a^ tumor suppressor protein (expressed from the *INK4a/ARF (Cdkn2a)* locus), which sequesters Cdk4 and Cdk6 and prevents their interaction with the D cyclins, increases with age in both rodent and human islets [34]. In the absence of p16^Ink4a^, Cdk4 and Cdk6 complex with Cyclin D and phosphorylate pRB, releasing the E2F transcription factor, which facilitates the G to S phase cell cycle transition. Increased p16^Ink4a^ is associated with cell cycle arrest and cellular senescence (see Figure 2) [32]. Beta cell-specific transgenic over-expression of p16 decreases beta cell proliferative capacity in young mice (26-32 weeks of age) to levels observed in older mice [34]. Conversely, germline deletion of *p16^Ink4a^* significantly ameliorates age-related decreases in beta cell proliferative capacity. In the absence of p16^Ink4a^, beta cells from mice at ~60 weeks of age showed levels of proliferation comparable to beta cells from younger mice (10 weeks of age). Proliferation still declined with age in mice lacking INK4a, but this was thought to be related to either the effect of p19^Arf^ expression or a mechanism that was independent of the products of the *Ink4a/Arf* locus [34]. p19^Arf^ (known as p14^ARF^ in humans) is the other product of the *INK4a/Arf* gene locus. This protein is also involved in cell cycle progression but by a different mechanism (see Figure 3). The function of this protein has been examined in the cancer and stem cell fields but little is known about its role in pancreatic islets. Interestingly, in other cell types, p19^Arf^ has been implicated as a direct inhibitor of FoxM1 [35].

The decline in beta cell proliferation with age may result in part from decreased expression of specific growth factor/hormone receptors or their downstream signaling components. For example, treatment with the glucagon-like peptide-1 (GLP-1) analog, Exendin-4 increases beta cell mass in young mice (6 weeks old) but not in mice aged 7-8 months, indicating that the aged beta cell does not respond very well to GLP-1 [36]. Exendin-4 treatment results in a marked decrease in p16^Ink4a^ levels in young but not old mice, reinforcing the concept that p16^Ink4a^ contributes to the reduced proliferative capacity of the older beta cell.

p16^Ink4a^ also plays a role in beta cell regeneration. Streptozotocin (STZ) causes beta cell necrosis and diabetes after a single, high dose injection in adult mice, and beta cell regeneration following destruction has been well-established [37]. *p16^Ink4a^* +/+, +/−, and −/− mice were injected with STZ, resulting in beta cell necrosis and diabetes in all genotypes. However, by 9-15 weeks of age *p16^Ink4a^* +/+ and +/− mice showed persistent hyperglycemia compared to their *p16^Ink4a^* −/− littermates, although even the p16^Ink4a^ deficient mice did not show complete recovery to pre-STZ levels of glucose tolerance, survival, and weight. These data once again highlight p16^Ink4a^-independent regulation of beta cell regenerative capacity with age [34].

#### Inhibiting the inhibitors

**Figure 4:**
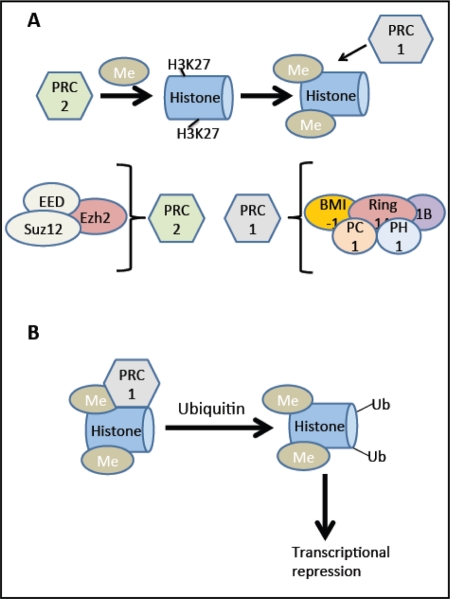
BMI-1 and Ezh2: Effects on transcription. **(A)** The Ezh2 (Enhancer of zeste homologue 2) component of the PRC2 (Polycomb repressor complex 2) complex methylates lysine (K) 27 of histone H3 (H3K27). This methylation process recruits the PRC1 (Polycomb repressor complex 1) complex. **(B)** The Ring1A and Ring1B components of this complex ubiquitylate H2AK119. This ubiquitylation causes transcriptional repression, for example at the *Ink4a/Arf* locus. In addition to Ezh2, the PRC2 complex contains EED (embryonic ectoderm development) and Suz12 (suppressor of zeste homologue 12). The PRC1 complex contains BMI-1, Ring 1A, Ring 1B, PH1 (Polycomb homologue 1), and PH1 (Polyhomeotic homologue 1). Adapted from [77].

Because inhibitors like p16^Ink4a^ reduce the proliferative capacity of the beta cell, inhibiting cell cycle inhibitors could offer an alternative way to circumvent this deficit. B-cell-specific Moloney murine leukemia virus integration site 1(Bmi-1) and Enhancer of zeste homologue 2 (Ezh2) are transcriptional regulators in the Polycomb family. Bmi-1 is part of the multiprotein histone E3 ubiquitin ligase complex, polycomb repressor complex 1 (PRC1; see Figure 4) and is important for maintaining the enzymatic activity of the complex as well as its structure [38], while Ezh2 is a component of PRC2 involved in methylation of lysine 27 of histone H3 (H3K27) and recruitment of PRC1. These complexes are both involved in transcriptional repression of cell cycle inhibitors including *p16^Ink4a^* and *p19^Arf^*, in the pancreas and other tissues.

Within the postnatal pancreas, Bmi-1 is localized to islets and its expression decreases significantly from 2 to 10 weeks of age [38]. Ezh2 is expressed in the beta cells of the pancreas, and its expression also decreases with age [39]. Using mice lacking Bmi-1, Dhawan et al. showed a significant increase in *p16^Ink4a^* mRNA as well as severely reduced Ki67 reactivity, a marker of proliferation, when comparing 2 week old mice to 10 week old mice [38]. These data show that Bmi-1 is critical to suppression of *p16^Ink4a^* and that beta cell proliferation is greatly reduced in the absence of its silencing. Mice with conditional gene inactivation of *Ezh2* in the beta cell showed a premature increase in *p16^Ink4a^* and *p19^Arf^* mRNA expression and reduced beta cell proliferation and mass, hypoinsulinemia, and mild diabetes in one month old mice. Interestingly, there was no change in mRNA levels of other *Ink4* or cdk inhibitors (*Ink4b, Ink4c, Ink4d, p21^Cip1^, p27^Kip1^, Trp 53*) suggesting that Ezh2 has a specific effect on the *INK4a/Arf* locus in the beta cell [39].

### The role of apoptosis in decreased beta cell mass

In a study of human pancreatic tissue by Reers et al., the decline in beta cell replication with age was not found to be associated with a change in the frequency of apoptosis [16]. In contrast, a study by Butler showed both a low frequency of beta cell replication and a higher rate of beta cell apoptosis in obese and diabetic individuals compared with lean and non-diabetic individuals [17]. Specifically, the non-diabetic obese individuals had a ~50% increase in relative beta cell volume (ie. beta cell area/exocrine area) compared to non-diabetic lean controls; obese individuals with impaired fasting glucose and Type 2 diabetes had a 40-63% beta cell volume deficit compared to obese non-diabetic controls. This indicates that those individuals with dysglycemia were unable to adaptively increase their beta cell volume. Ki67 (a marker of proliferation) labeling of the pancreata showed that all individuals had a low frequency of replication, although there was a trend toward decreased beta cell replication with age. When normalized for beta cell mass, there was a three-fold and ten-fold increase in beta cell apoptosis in obese and lean diabetic individuals compared to obese and lean non-diabetic individuals respectively as observed by TUNEL staining [17]. This balance of replication and apoptosis is important to remember when considering whether the decline in beta cells in the elderly is truly an issue of inadequate proliferation or whether there is also elevated apoptosis. These studies indicate that in the non-diabetic individuals, even when controlling for obesity, which causes higher rates of apoptosis, enhancing beta cell replication alone may be inadequate.

#### Amylin aggregation

Islet amyloid polypeptide (IAPP) or amylin [40] is a thirty-seven amino acid neuroendocrine hormone that is co-secreted with insulin from the beta cell [41]. Amylin suppresses glucagon secretion and in conjunction with insulin helps to regulate glucose homeostasis [42]. In Type 2 diabetics, hypersecretion of insulin results in increased co-secretion of amylin. This amylin aggregates into amyloid plaques, which can subsequently lead to increased beta cell apoptosis and cause progression of diabetes [43]. Amyloid plaques are present in >90% of Type 2 diabetics at autopsy [44].

With age there is an increased deposition of amylin in the islets of diabetic individuals but not non-diabetic individuals as seen by examination of pancreata at autopsy [45]. Interestingly, rodent amyloid does not aggregate due to a proline amino acid substitution that makes the rodent IAPP differ from human IAPP (hIAPP) [46]. Therefore, to study the effects of amylin aggregation on the beta cell, transgenic mice were developed that expressed human (h)IAPP under control of the insulin promoter. Over-expression of hIAPP resulted in hyperglycemia in male mice aged 6 to 9 months without the presence of amyloid plaques. In older (>13 month) transgenic male mice, there was a significant amount of amylin plaque formation in the peripheral and perivascular areas of the islets only [47]. Hyperglycemia preceded the formation of obvious plaques, suggesting that the hyperglycemia could be, at least in part, due to cytotoxicity from intermediate-sized amyloid particles. Janson et al. showed that when applying freshly dissolved hIAPP exogenously to dispersed mouse and human islets, islet cell apoptosis and necrosis occurred within 24 to 48 hours. In contrast, when islets were treated with large amyloid deposits, there was no observable effect. Further study showed that the intermediate-sized amyloid particles caused membrane damage and subsequent cell death [48]. From these data, it is clear that increases in amylin deposition size can cause increased beta cell death and progression of Type 2 diabetes, however the size of the particles may be critical to determining whether they have detrimental effects. These results demonstrate that increased rates of beta cell apoptosis, in the absence of any known defect in proliferation capability, can lead to reduced beta cell mass.

### Mechanisms for increasing beta cell mass, regeneration or neogenesis

As discussed above, beta cell proliferation decreases with age, but how do beta cells adapt and increase their beta cell mass? FoxM1 is important to beta cell replication, and its role in tissue regeneration was first appreciated using partially hepatectomized mice. Transgenic restoration of FoxM1 expression in older hepatocytes to levels similar to those found in young mice resulted in improved liver regeneration in older mice [12]. Genetic inactivation of the *Foxm1* gene throughout the pancreatic epithelium results in a reduced ability of beta cells to respond to proliferative stimuli and impaired beta cell regeneration following partial pancreatectomy [13-15]. Studies from the Kushner lab revealed that beta cell mass expansion in response to multiple different stimuli (partial pancreatectomy, beta cell destruction, and GLP-1 analog) severely declines with age [49], possibly due to the decrease in *Foxm1* expression.

It has become clear in recent years, at least in rodent models, that increases in beta cell number postnatally occur mainly through proliferation of existing beta cells, with little to no contribution from neogenesis from stem or progenitor cells [50] [51]. Taken together, these studies suggest that the loss of proliferative capacity of existing beta cells, rather than the loss of progenitor cells, is likely cause of reduced beta cell mass expansion in older individuals.

Pregnancy is a condition under which there is an increased demand for insulin production and secretion, although the mechanism by which this occurs in humans may differ from that of rodents. An inadequate insulin response during pregnancy results in gestational diabetes. During pregnancy, rodents exhibit both beta cell hyperplasia and hypertrophy [52]. In contrast, a review of 44 human pancreata from pregnant, post-partum, and non-pregnant women at autopsy suggested that the increase in insulin-positive area in pregnant and post-partum women was most likely due to islet neogenesis rather than proliferation [53]. Surrogate markers for neogenesis include the number of insulin-positive cells within or near pancreatic ducts and single insulin-positive cells scattered throughout the exocrine pancreas. However, the role of proliferation versus neogenesis in pregnant human females is still controversial as studies from Van Assche et al. concluded that beta cells undergo both hyperplasia and hypertrophy during pregnancy [54]. These differences may be accounted for by a smaller study population (5 pregnant and 5 non-pregnant women) in the Van Assche study, differences in techniques used (Ivic's Victoria blue acid fuchsin staining vs. insulin immunohistochemistry to ascertain fractional endocrine and beta cell area), and underlying disease conditions that may have influenced the beta cell. Thus, in contrast to the rodent model, in human pregnancy, an increase in beta cell number may result from mechanisms other than increased beta cell replication, perhaps islet neogenesis. It is important to note though that the rodents used in the pregnancy studies were young adults, in contrast to the subjects in published human studies. Younger individuals would be expected to demonstrate increased beta cell replication and hypertrophy during pregnancy; epigenetic changes in beta cells that occur with age likely result in decreased response of beta cells to replication cues in both rodents and humans with age [55].

As previously mentioned, beta cell loss may be accompanied by a reduction in islet neogenesis or beta cell proliferation, and/or increased apoptosis. To elucidate the rate of human beta cell turnover, examination of ten human cadaver pancreata was undertaken. These ten individuals had received iododeoxyuridine (IdU) or bromodeoxyuridine (BrdU) from eight days to four years prior to death during cancer treatment clinical trials. Radiocarbon dating and in vivo thymidine analog staining showed that only individuals under the age of thirty years showed evidence of beta cell turnover. This implies that therapies directed at beta cell expansion may not be as effective in individuals over the age of thirty [56].

Since pregnancy is a physiological condition during which there is an appropriate response to increased insulin demand, this is an excellent model to investigate the mechanisms by which insulin production or beta cell mass could be increased in adults. Although there is a consistent increase in beta cell mass observed in both rodents and humans during pregnancy, the mechanism(s) underlying this expansion have not yet been fully elucidated and may differ significantly in rodent and human pregnancy. From a limited examination of human pancreata, it seems that beta cell turnover is severely diminished in individuals older than thirty and thus, strategies to improve the receptivity of older beta cells to proliferative cues should be investigated.

### Factors regulating beta cell function and maintenance of differentiation.

#### Pdx1 and MafA

Pdx1 (pancreatic and duodenal homeobox 1), also known as IDX1, IPF1, STF1, and GSF, is a transcription factor critical for beta cell development and function. Mice with late onset beta cell-specific *Pdx1* inactivation displayed approximately 60% of the normal number of beta cells and 10% of total pancreatic insulin content compared to wild type mice at three weeks of age. Additionally, these mice had an increased number of glucagon-expressing cells as compared to wild type, with 22% of the glucagon- or insulin-expressing cells co-expressing both hormones [57]. Homozygous *pdx1* inactivation during embryogenesis results in early onset diabetes due to decreased beta cell mass and increased alpha cell area at birth [18] while a heterozygous inactivation results in persistent hyperglycemia with a relative deficiency of plasma insulin. Although beta cell mass was unchanged, non-beta cell islet mass was nearly doubled suggesting that the heterozygous inactivation of *Pdx1* leads to impairments in glucose homeostasis [58]. Supporting this conclusion, islets from *pdx1* heterozygous mice released approximately 45% less insulin in response to a glucose stimulus compared to wild-type islets [59]. These data show the importance of Pdx1 in maintaining the beta cell phenotype in the adult mouse and repressing glucagon expression in insulin-positive cells.

Seven to eight month old rats show a decreased number of insulin-positive cells that express Pdx1 [60], and 56% and 33% reduction in *pdx1* mRNA levels in 22 month old compared to 2 month old rats and mice respectively [61]. Acute deletion of *Pdx1* at 3 months results in an acute reduction of Pdx1 but does not cause any changes in glucose homeostasis. A doxycycline-inducible transgenic system in which expression of an antisense ribozyme that cleaves the *pdx1* mRNA at a specific site, leading to a reduced Pdx1 protein level, was utilized to examine the effect of reduced Pdx1 function in older vs. younger mice. After 3 weeks of doxycycline treatment, 18 month old transgenic mice had significantly worse glucose tolerance than age-matched controls [62]. It can thus be concluded that *Pdx1* expression declines with age in mouse beta cells, that this contributes to reduced beta cell function, and that beta cell function is particularly affected by reduced *pdx1* levels with age.

MafA is a beta cell-restricted transcription factor, which in combination with transcription factors Pdx1 and Beta2 synergistically activates the insulin promoter [63]. The effect of age on MafA expression levels has not yet been studied, but it has been shown that MafA null mutant mice, though normoglycemic at birth, develop diabetes by four weeks of age [64]. This suggests that MafA may be important to maintaining glucose homeostasis as animals age. It is therefore possible that with age individuals who develop diabetes could have some disruption in MafA levels as it is already known that in diabetes, MafA levels are reduced [65, 66]. Glucose toxicity causes reduced insulin gene expression [67], perhaps due to reduced MafA and Pdx1 levels, the loss of MafA precedes the reduction of Pdx1 [68]. Restoring MafA and Pdx1 levels to pre-glucotoxic levels virtually completely rescues insulin mRNA levels [69].

#### Antioxidants

Oxidative stress increases with age [70] in many tissues, and this is probably also true in the beta cell. Glucose toxicity increases intraislet peroxide levels [71] and treatment with antioxidants improves glucose levels by reducing apoptosis rates [72] and improving insulin gene expression, insulin secretion, and Pdx1 binding to the insulin promoter [73]. In the db/db obese mouse model, it has been shown that reversing beta cell oxidative stress by glutathione peroxidase over-expression, restored MafA expression and subsequently improved beta cell volume and glucose homeostasis [74].

## CONCLUSION

An increased incidence of diabetes is observed with age, and there are many possibly reasons for this. One of these is that the beta cell has reduced proliferative capacity and in diabetic individuals this is further confounded by higher rates of beta cell apoptosis. The currently known underlying mechanisms behind the reduction in beta cell proliferation observed with age include reduced expression of cell cycle activators, increased expression of cell cycle inhibitors, reduced *pdx1* expression, and increased amylin aggregation. Studying aging in the non-diabetic rodent and human models is currently a developing field; therefore very few broad conclusions can be drawn. Further study in these areas is important as they could indicate targets for preventing or slowing the progression of diabetes with age.
